# Physiological Testing of Drugs

**Published:** 1898-06

**Authors:** 


ARTICLE V.

### PHYSIOLOGICAL TESTING OF DRUGS.

The physiological activity of medicinal agents is just now receiving much attention from physicians, and methods have been devised whereby it can be directly tested, directly apart from chemical analysis. In the near future, if we are to believe the *Pharmaceutical Era*, April 15th, pharmacists may be required to guarantee not only that the drugs they sell are pure, but that they possess the required ability to act on the systems of those to whom they may be administered. Says the *Era* :

"It is well known among investigators that there are certain classes of remedies which can not be standardized by any chemical means. Some of these remedies are the most important in the whole Pharmacopeia. The group of heart tonics is perhaps the most notable, including as it does a very large number of drugs that are constantly prescribed by the physician. Of this group of heart tonics, undoubtedly, digitalis is the most important."

In what follows, tests of the action of this drug on various parts of the animal circulation are described. We quote below only that for studying the direct action of the drug on the heart.

"For the purpose of studying the action of the drug upon the frog's heart more closely; a frog is taken (which has had its brain destroyed), fastened in a dorsal position to a small piece of board, and the thorax opened by a long median incision and the heart exposed, being careful to avoid woundlng the blood-vessels. The heart is seen beating in the thin transparent pericardial sac in the space between the two lungs. The rhythmic and alternate contraction and expansion of the auricles and ventricle (the frog's heart consists of two auricles which open into a single ventricle, whereas in the mammalian heart we have two auricles and two ventricles), occuring about thirty times per minute, is a beautiful illustration of the action of this most vital organ. The red dilated ventricle quickly contracts and becomes reddish-gray. Let us put a drop of dilute solution of digitalis upon the beating heart and await the result. It soon begins to beat more slowly, * * * the ventricle relaxes less and less, the red color becomes less and less intense, and the heart expands with greater and greater difficulty. Soon there are two beats of the auricle to one of the ventricle, and then three to one, and so on until finally the ventricle makes one final effort and ceases to beat entirely, * * * notwithstanding the continued efforts of the auricles to force the blood into the chamber. Soon, however, the auricles cease to beat also. If we examine the ventricle of the heart carefully after it has ceased beating and compare its appearance with that presented by the ventricle after the normal systole of the heart, we find that it is smaller and much lighter colored, the reddish tint having almost entirely disappeared. Its internal cavity has been entirely eliminated by the drawing together of its muscular fibers. If desired we can take a graphic tracing from the heart on the kymographion by nicely balancing a straw lever, tipped with a piece of tin, in such a way that it rises and falls as the heart alternately contracts and expands, the record being marked by the point of the lever on a revolving drum covered with a sheet of smoked paper."

It is evident that such an experiment as this furnishes a delicate test of the activity of the drug, and that the test may be recorded permanently by means of the tracing. The day may come, perhaps, when a properly certified tracing of this kind will accompany every bit of digitalis ordered by a retail druggist from a wholesale dealer.—*Dental Register.*

